# Interrelations of phantom ringing related anxiety and personal self-esteem in undergraduate university students

**DOI:** 10.1192/j.eurpsy.2024.873

**Published:** 2024-08-27

**Authors:** E. L. Nikolaev, M. Alhasan

**Affiliations:** ^1^Department of Social and Clinical Psychology; ^2^Medical Faculty, Ulianov Chuvash State University, Cheboksary, Russian Federation

## Abstract

**Introduction:**

Manifestations of phantom ringing syndrome are widely seen in healthy population. Are there any interrelations between this phenomenon and personal psychological characteristics that are connected with the level of their mental health?

**Objectives:**

To determine the specificity of interrelations of phantom ringing syndrome related anxiety and personal self-esteem in university students

**Methods:**

The anonymous survey covered 546 undergraduate university students. The questions were centered on the students’ patterns of their personal smartphone use.

**Results:**

The research showed that manifestations of phantom ringing syndrome is available in 189 students, or in every third student (34.6%), who use mobile phones. It is equally represented in males (49.7%) and females (50.2). Clinically, it is characterized by a higher level of anxiety, which reliably correlates (p<0.01) with the level of stress (r=.17), level of nervousness caused by absence of a mobile phone (r=.18), the frequency of headache (r=.15), the frequency of medication consumption related to chronic somatic disease (r=.15). We also established valid negative interrelations between the level of phantom ringing syndrome related anxiety and the personal self-esteem based on the parameters of religious belief (r=-.15), personal attractiveness (r=-.16), mind (r=-.17), happiness (r=-.24), liveliness (r=-.25) and well-being (r=-.15). We have not found any proof of valid interrelations with self-assessment of health.

**Conclusions:**

The received results prove that phantom ringing syndrome related anxiety is connected with the personal self-esteem, the level of the perceived stress and some other clinical manifestations

**Disclosure of Interest:**

None Declared

